# Research Progress on the Surface of High-Nickel Nickel–Cobalt–Manganese Ternary Cathode Materials: A Mini Review

**DOI:** 10.3389/fchem.2020.00761

**Published:** 2020-08-28

**Authors:** Liubin Song, Jinlian Du, Zhongliang Xiao, Peng Jiang, Zhong Cao, Huali Zhu

**Affiliations:** ^1^Hunan Provincial Key Laboratory of Materials Protection for Electric Power and Transportation, School of Chemistry and Food Engineering, Changsha University of Science and Technology, Changsha, China; ^2^School of Physics and Electronic Science, Changsha University of Science and Technology, Changsha, China

**Keywords:** lithium ion battery, high nickel type, ternary cathode material, surface, core-shell

## Abstract

To address increasingly prominent energy problems, lithium-ion batteries have been widely developed. The high-nickel type nickel–cobalt–manganese (NCM) ternary cathode material has attracted attention because of its high energy density, but it has problems such as cation mixing. To address these issues, it is necessary to start from the surface and interface of the cathode material, explore the mechanism underlying the material's structural change and the occurrence of side reactions, and propose corresponding optimization schemes. This article reviews the defects caused by cation mixing and energy bands in high-nickel NCM ternary cathode materials. This review discusses the reasons why the core-shell structure has become an optimized high-nickel ternary cathode material in recent years and the research progress of core-shell materials. The synthesis method of high-nickel NCM ternary cathode material is summarized. A good theoretical basis for future experimental exploration is provided.

## Introduction

As environmental issues have become a major concern, reducing the use of fossil fuels has become a key issue. Lithium-ion batteries are the most commonly used energy storage devices due to their high energy density and long cycle life (Wang et al., 2020f; Zhang et al., 2020). The new energy industry powered by lithium-ion batteries has been greatly developed (Pant and Dolker, 2017; Barcellona and Piegari, 2020; Mossali et al., 2020; Wang et al., 2020d). However, fierce competition in this industry has brought about higher requirements for lithium-ion batteries (Zubi et al., 2018). The nature of the electrode material is the fundamental factor affecting the performance of the battery. Analyzing and optimizing the electrode material is an important approach to solving the bottleneck of the lithium ion battery (Lipu et al., 2018; Zhang et al., 2018).

LiCoO_2_ has good cycle stability in the cathode material of lithium batteries, but the actual capacity is low (Yang et al., 2018; Wang et al., 2020b). LiMn_2_O_4_ has excellent cycle performance but is prone to spinel phase degradation (Dai et al., 2012; Bhuvaneswari et al., 2019). LiMnO_2_ has good cycle performance but low preparation efficiency (Zheng et al., 2016; Zhou et al., 2016). LiNiO_2_ has high energy density but is prone to structural disorder (Liu et al., 2007; Deng et al., 2019). A layered lithium nickel–cobalt–manganese (NCM) oxide LiNi_x_Co_y_Mn_z_O_2_ (LNCM) ternary cathode material with the combined advantages of LiCoO_2_, LiNiO_2_, and LiMnO_2_ has been generated (Park and Choi, 2018). In LNCM, the valences of nickel, cobalt, and manganese cations are usually +2, +3, and +4, respectively (Kang et al., 2006; Lin et al., 2014). Among them, +4 valence Mn guarantees structural stability, whereas +3 valence Co regulates cationic disorder and reduces surface energy (Garcia et al., 2017). The redox couple energy of Ni^2+/4+^ and Co^3+/4+^ can increase the battery's capacity (Lee et al., 2014). According to the crystal field theory, Ni mostly exists in the form of +2 valence. The radius of Ni^2+^ is close to the radius of Li^+^. Cation mixing easily occurs in high-nickel type NCM ternary cathode materials. Ni^4+^ has strong oxidizability; Li_1−x_NiO_2_ formed after delithiation has poor thermal stability; Ni^4+^ easily reacts with organic electrolyte (Hong et al., 2012). The main lattice of the highly delithiated electrode surface releases oxygen, which reacts with the organic electrolyte (Abraham et al., 2002). The surface of the high-nickel material reacts with the external CO_2_ and H_2_O to form a lithium-containing compound (Liu et al., 2016; Gao et al., 2019).

This review starts with the surface and interface of the high-nickel NCM ternary cathode material. The causes of the defects in the material are analyzed. The core-shell structure that improves the performance of the high-nickel NCM ternary cathode material is explained. The methods of generating high-nickel-type NCM ternary cathode material are mentioned.

## Study on the Surface and Interface Structure of High-Nickel Nickel–Cobalt– Manganese Ternary Cathode Materials

The stable electrode surface and interface structure are the key factors that determine the quality of the battery. Structural defects and side reactions on the surface of the high-nickel NCM ternary positive material affect the transfer of electrons and the deintercalation of lithium ions, thereby affecting the performance of the battery (Wang et al., 2020c). The changes in the chemical properties of lithium-ion batteries in terms of surface and structure need to be elucidated.

### Surface Structure and Evolution of High-Nickel Cathode Materials

The high-nickel NCM ternary cathode material has a-NaFeO_2_ structure; the space group is hexagonal R-3m; Li^+^ is embedded in the layered structure of transition metal and oxygen atoms and inserted and extracted in the 2D gap (Li et al., 2014; Liu Y. et al., 2019). In high-nickel type LNCM cathode materials, Ni^2+^ and Li^+^ are prone to cation mixing (Yang et al., 2019b). Cation mixing shifts the hierarchical R-3m space group to the tightly packed spinel Fm-3m space group. This tight structure leads to shorter ion spacing and larger interactions, making Li^+^ diffusion difficult (Zhang et al., 2017). Studies have suggested that structural changes occur on the surface of high nickel layered oxides (Li J. et al., 2020; Liu et al., 2020).

**Figure 1:**
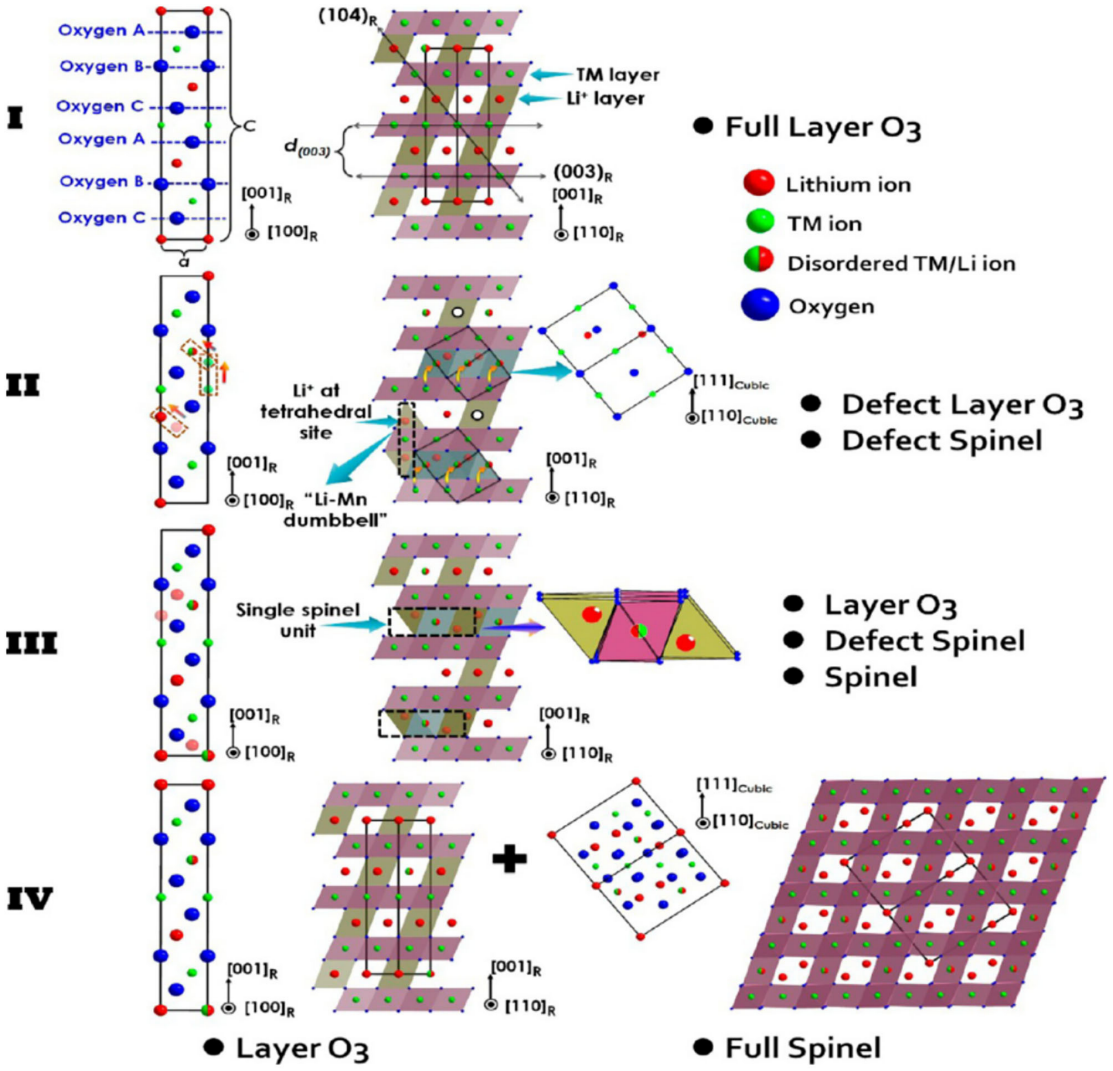
High-nickel cathode material in four-stage microstructure of the atomic distribution and order of the schematic explanation.

In layered NMC materials, Li^+^ jumping and migration barriers are very sensitive to local structures (Van der Ven and Ceder, 2001; Kang and Ceder, 2006). Based on this, the diffusion rate of Li^+^ in LiNi_0.8_Mn_0.1_Co_0.1_O_2_ (NMC811) is found to be the main reason for the structural change of the material. When the degree of lithiation deepens, the volume of a single positive electrode particle continues to shrink after delithiation. The lattice parameters change along with the material structure (Märker et al., 2019). Fu et al. (2014) found that with increasing number of lithium sources, the lattice parameters (a and c) and the thickness of the intercellular space decrease, and the Li^+^/Ni^2+^ mixed arrangement causes structural changes. Wang et al. (2017) studied the LiNi_0.6_Co_0.2_Mn_0.2_O_2_ (NMC622) material and found that spinel skeleton defects and a sharp drop in lattice parameter c cause lattice distortion. Moreover, the spinel structure causes the instability of the material surface and structure. Figure 1 shows the processing of high nickel NCM ternary cathode material, which is due to cation mixed discharge caused by structural changes (Wang et al., 2017). Many studies believe that heterogeneous ions can be inserted into the lattice through doping, thereby changing the bond energy and lattice parameters and suppressing the deterioration of the internal structure of the lattice (Binder et al., 2018; Yu et al., 2020).

### Mechanism of Surface Redox of High-Nickel Nickel-Cobalt-Manganese Ternary Cathode Material

The thermal decomposition products of high-nickel LNCM cathode materials at high temperature may include the following: Li_x_Mn_2_O_4_, LiNiO_2_, (NiO)_x_(MnO)_y_, CoO, CoCO_3_, LiF, and various oxides of manganese, nickel, and cobalt (Wang et al., 2020a). The electronic structure of the active element in the transition metal layer is a factor that directly affects the redox reaction. Co and Ni have a +3 valence, and the energy band is (t_2g_)^6^(e_g_)^0^, which is in a low spin state. The release of more than half of Li^+^ ions from the layered LiCoO_2_ will cause O^2−^ 2p electron loss. The top of the O^2−^ 2p band overlaps with the t_2g_ band with Co^3+/4+^ redox activity, resulting in structural instability. The overlapping position of the e_g_ band of Ni^3+/4+^ and the top of O^2−^ 2p band is lower than that of the t_2g_ band of Co^3+/4+^, indicating that the delocalization effect of Ni^3+/4+^ is smaller, the structure is more stable, and the reversible performance is better (Hou et al., 2017). The e_g_ band of Mn does not overlap with O^2−^ 2p, and the overlap of the t_2g_ band and O^2−^ 2p is lower than that of Co^3+/4+^. Thus, the stability of Mn is higher. Julien et al. (2014) established a ternary LiNi_y_Mn_y_Co_1−2y_O_2_ oxide with better stability on the basis of LiNiO_2_ and LiCoO_2_ oxide structures (Julien et al., 2014).

Many studies have hoped to influence the energy band through doping and thereby improve the material's stability. Common doping approaches are as follows: anion doping: F^−^ (Zhao et al., 2019), Cation doping: K^+^ (Liu Z. et al., 2019), Al^3+^ (Trease et al., 2016; Do et al., 2018), Zr^4+^ (Sivaprakash and Majumder, 2009), Mg^2+^ (Jin et al., 2019), Ti^4+^ (Zhang et al., 2019), Co-doping: Mn^4+^-${\text{PO}}_{4}^{3-}$ (Qiu et al., 2019), and Al^3+^-Mg^2+^ (Woo et al., 2009). However, the doping of foreign elements can cause structural collapse because of the doping ions' inability to integrate into the layered structure.

### Side Reaction of High Nickel Nickel-Cobalt-Manganese Ternary Cathode Material Interface Structure

High-nickel LNCM cathode materials are prone to side reactions at the interface with the electrolyte. Side reactions and the products of such reactions can affect battery performance. Usually, the decomposition of the electrolyte is as follows (Van Ree, 2020):

(1) $$\begin{array}{l} \text{In general}:\text{ LiP}{\text{F}}_{6}\;\left(\text{s}\right)↔\text{LiF}\left(\text{s}\right)+\text{P}{\text{F}}_{5}\;\left(\text{g}\right) \end{array}$$

(2) $$\begin{array}{l} \text{In the presence of }{\text{H}}_{2}\text{O}:\text{P}{\text{F}}_{5}+{\text{H}}_{2}\text{O}\to \text{PO}{\text{F}}_{3}+2\text{HF} \end{array}$$

(3) $$\begin{array}{l} 2\text{PO}{\text{F}}_{3}+3\text{L}{\text{i}}_{2}\text{O}\to 6\text{LiF↓}+{\text{P}}_{2}{\text{O}}_{5}\text{↓}\left(\text{or L}{\text{i}}_{\text{x}}\text{PO}{\text{F}}_{\text{y}}\right) \end{array}$$

The high-nickel type LNCM positive electrode easily reacts with the surrounding environment due to its high surface reactivity (Jung et al., 2018). LiF, Li_2_CO_3_, LiOH, and other impurities are easily deposited on the interface between the active high nickel LNCM positive electrode and the electrolyte, thereby suppressing the diffusion of Li^+^ and reducing the electrochemical performance. To effectively prevent the side reaction between the electrode and the electrolyte, coating modification is proposed, such as coating metal oxides: Al_2_O_3_ (Liao and Manthiram, 2015; Yan et al., 2016), ZrO_2_ (Yang et al., 2019a), MgO (Yoon et al., 2012), ZnO (Chang et al., 2010), lanthanide oxides: La_4_NiLiO_8_ (Li L. et al., 2020), phosphate: AlPO_4_ (Zhao et al., 2017), Cu_3_(PO_4_)_2_ (Zhao et al., 2016), fluoride: AlF_3_ (Ding et al., 2017), transition metal oxide: Li_2_ZrO_3_ (Xu et al., 2016), multiple coating: Li_2_${\text{TiO}}_{3}^{\text{\_}}$Li_2_ZrO_3_ (Li J. et al., 2020), and ${\text{LiFePO}}_{4}^{\text{\_}}$Al_2_O_3_ (Seteni et al., 2017). The double modification method combines doping and coating, as follows: Sr doping–LaMnO_3_ coating (Li et al., 2019), N doping–C coating (Nanthagopal et al., 2019), Zr doping–ZrO_2_ coating (Wang et al., 2020e), and Sn doping–Li_2_SnO_3_ coating (Zhu et al., 2020).

## Study on High-Nickel Nickel–Cobalt–Manganese Ternary Cathode Materials With Core-Shell Structure

The high-nickel type NCM cathode material is a combination of three transition metal elements. This material does not solve the defects of any one element. Although element doping, surface coating, and double modification can improve defects, these solutions only involve the simple processing of the body material and do not fundamentally solve the problem. Sun et al. (2005) extended the concept of cladding to the core shell and proposed the concept of using the core shell material for lithium ion batteries. The high-nickel nickel–cobalt–manganese ternary cathode material with a core-shell structure has evolved from a common core-shell structure to a core-shell gradient structure, and finally, to a full gradient core-shell structure.

### Simple Core-Shell Structure

In the high-nickel type NCM cathode material with a simple core-shell structure, a synergistic effect exists between the core and the shell. The core material has high specific capacity performance, and the shell material has structural and thermal stability. Sun et al. (2006b) used Li[Ni_0.8_Co_0.2_]O_2_ with high specific capacity as the core and Li[Ni_0.5_Mn_0.5_]O_2_ with high structural stability as the shell. They obtained a simple core-shell Li[(Ni_0.8_Co_0.2_)_0.8_(Ni_0.5_Mn_0.5_)_0.2_]O_2_ cathode material. Compared with the Li[Ni_0.8_Co_0.2_]O_2_ electrode, the capacity retention rate and thermal stability of the abovementioned synthesized cathode material are significantly improved. Shi et al. (2014) synthesized Li[(Ni_0.8_Co_0.1_Mn_0.1_)_0.7_(Ni_0.45_Co_0.1_Mn_0.45_)_0.3_]O_2_ with (Ni_0.8_Co_0.1_Mn_0.1_)_0.7_ as the core and (Ni_0.45_Co_0.1_Mn_0.45_)_0.3_ as the shell. The core-shell material cyclicity and thermal stability showed significant improvement. Jun et al. (2017) used LiNiO_2_ as the core and Li[Ni_0.8_Co_0.1_Mn_0.1_]O_2_ as the shell to obtain Li[Ni_0.95_Co_0.025_Mn_0.025_]O_2_ core-shell material, which provided excellent discharge capacity while exhibiting excellent cyclic performance. The simple core-shell structure effectively improves the performance of the battery, but the composition of the core and shell materials in this structure is significantly different, thereby producing a large interface resistance and hindering Li^+^ diffusion. The high-temperature calcination process easily causes metal ion diffusion, resulting in structural changes in the material.

### Concentration Gradient Core-Shell Structure

**Figure 2:**
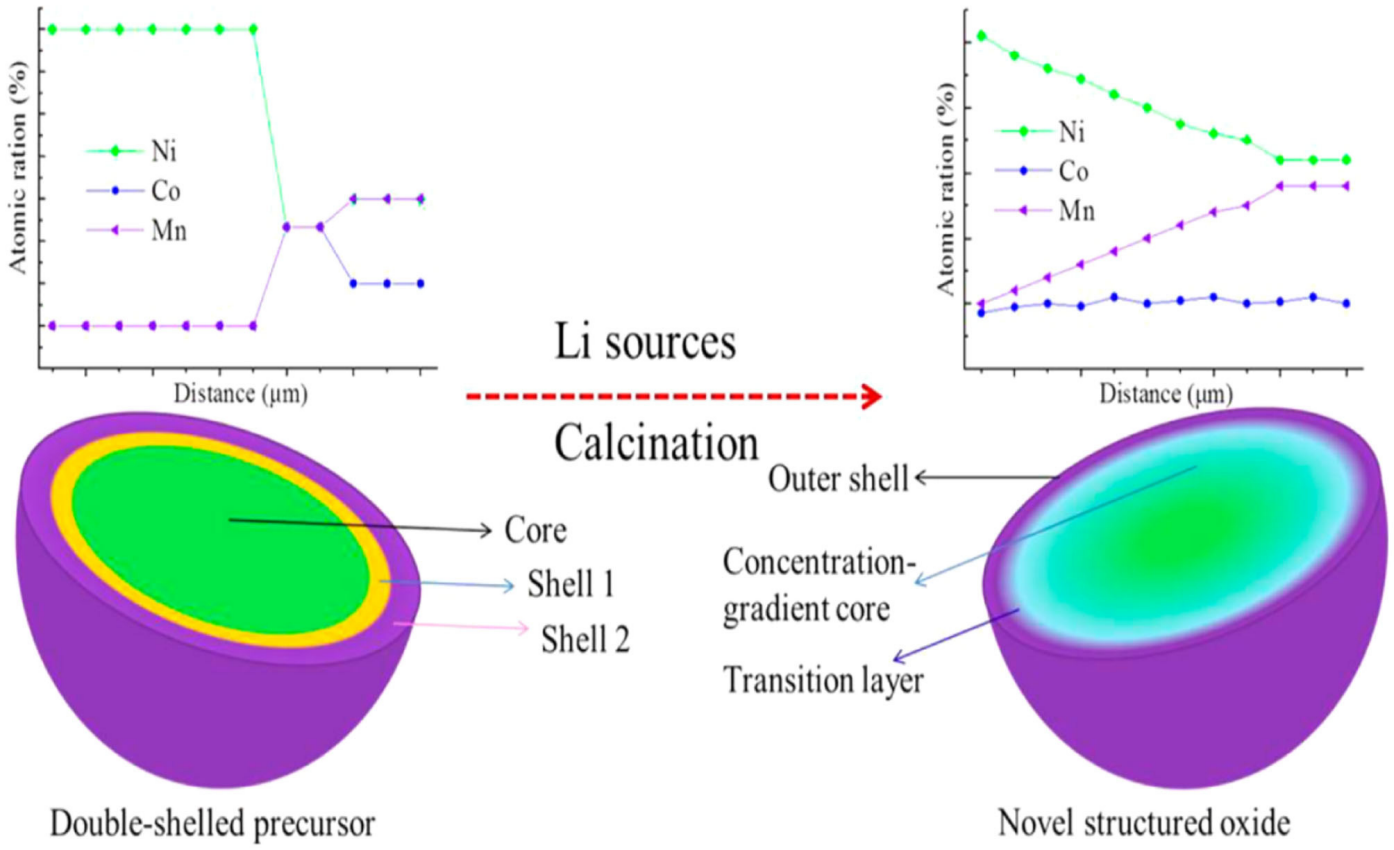
Schematic diagram of using a multi-shell precursor to control a positive electrode with a high nickel concentration gradient.

The concentration gradient core-shell structure is a new concept. It is proposed on the basis of a simple core-shell structure. A high-nickel-type NCM ternary material is coated with a shell material whose nickel concentration continuously decreases from the inside out. Liao et al. (2016) obtained the concentration gradient of the LiNi_0.76_Co_0.1_Mn_0.14_O_2_ cathode material from the double-shell [Ni_0.9_Co_0.1_]_0.4_[Ni_0.7_Co_0.1_Mn_0.2_]_0.5_[Ni_0.5_Co_0._1Mn_0.4_]_0.1_(OH)_2_ precursor's sintering, which significantly improves the capacity retention rate. Song et al. (2015) synthesized a concentration gradient LiNi_0.5_Co_0.2_Mn_0.3_O_2_ material. Figure 2 shows the principle of sintering a concentration gradient positive electrode material from a double-shell precursor (Song et al., 2015). The concentration gradient of the CG-LiNi_0.7_Co_0.15_Mn_0.15_O_2_ cathode material is prepared from the multilayer precursor, which effectively reduces side reactions and rapid Li^+^ kinetics (Hou et al., 2018). With the concentration gradient of the Li_1.2_Ni_0.13_Mn_0.54_Co_0.13_O_2_ cathode material, the initial reversible capacity and capacity retention rate are improved (Ma et al., 2019). Liao and Manthiram (2015) used a concentration gradient [Ni_0.2_Mn_0.8_]_0.3_ shell to encapsulate a high nickel [Ni_0.8_Co_0.2_]_0.7_ core and coated Al_2_O_3_ on the surface of the shell to obtain a sample with better cyclic stability, rate performance, and thermal stability. The concentration gradient core-shell structure has a shell material with continuous concentration changes, which effectively reduces the interface resistance between the core and the shell and strengthens the synergistic effect between the core and the shell. However, the final surface of this structure still has a relatively high Ni content and a relatively low Mn content. When the high-temperature and high-voltage states are cycled for a long time at a high ratio, the electrochemical performance deteriorates.

### Full Concentration Gradient Core-Shell Structure

The full concentration gradient core-shell material refers to the entire ternary precursor cathode material from the inside to the outside. The Ni ion content gradually decreases, and the Mn and Co ion contents increase continuously. The structure abandons the concept of having a clear core-shell interface and overcomes the problem of uneven coating of the shell layer or the large difference between the shell and core components. Ju et al. (2013) synthesized a full concentration gradient core-shell material from the center of the particle (Ni is 0.62–0.74 mol%; Co is 0.05 mol%) to the surface (Ni is 0.48–0.62 mol%; Co is 0.18 mol%). FCG-Li[Ni_0.59_Co_0.16_Mn_0.25_]O_2_ cathode material has a maximum discharge capacity of 188 mAh·g^−1^. The FCG cathode material that gradually changes from Li[Ni_0.86_Co_0.07_Mn_0.07_]O_2_ at the center of the particle to Li[Ni_0.67_Co_0.09_Mn_0.24_]O_2_ at the surface shows high capacity performance (Noh et al., 2014). Full concentration gradient core-shell material LiNi_0.7_Co_0.10_Mn_0.2_O_2_ has higher cycle performance and high temperature stability and rate performance (Liang et al., 2015). The relative molar content of Ni in the full concentration gradient core-shell material LiNi_0.8_Co_0.1_Mn_0.12_ cathode material gradually decreases from 84% to 76%, the relative molar content of Mn gradually increases, and the Co content shows a slow gradient variation. The capacity retention rate of this material after 100 cycles at 5C rate reaches 90% (Jiang et al., 2019).

## Method For Synthesizing High-Nickel Nickel–Cobalt–Manganese Ternary Cathode Material

Different synthesis methods will affect the microstructure and electrochemical performance of the prepared materials. At present, the methods for preparing the high-nickel nickel-cobalt-manganese ternary cathode material for lithium ion batteries mainly include the co-precipitation and high-temperature solid phase methods.

A material synthesized by the co-precipitation method has a small and uniform particle size and is typically used for coating the high-nickel NCM ternary cathode material and for the synthesis of the core-shell structure. For example, the following are prepared by co-precipitation method: LiNi_0.6_Mn_0.2_Co_0.2_O_2_ (Ren et al., 2017), LiNi_0.8_Mn_0.8_Co_0.1_O@Li_3_PO_4_@PPy (Chen S. et al., 2017), Li[(Ni_0.8_Co_0.1_Mn_0.1_)_1−x_(Ni_0.5_Mn_0.5_)_x_]O_2_ (Sun et al., 2006a), LiNi_0.8_Co_0.1_Mn_0.1_O_2_@x[Li-Mn-O] (Li et al., 2018), and LiNi_0.8_Co_0.1_Mn_0.1_O_2_@active material core-shell material (Su et al., 2019). High temperature solid phase method is typically used for doping modification, as follows: Ca doping LiNi_0.8(1−x)_Co_0.1_Mn_0.1_Ca_0.8x_O_2_ (Chen M. et al., 2017), Mn doping LiNi_0.82−x_Co_0.12−x_Mn_0.06+2x_O_2_ (Cho et al., 2018), and Mo doping LiNi_0.6_Co_0.2_Mn_0.2_O_2_ (Xue et al., 2018). Sol-gel, hydrothermal, and spray drying methods, as well as other preparation methods, are also available. Sol-gel method is used to prepare γ-Al_2_O_3_-coated NCM622 (Wu et al., 2019) and tungsten oxide-coated NCM-811 (Becker et al., 2019). LiNi_0.7_Co_0.15_Mn_0.15_O_2_ is prepared by hydrothermal method (Tian et al., 2015). NCM811 is prepared by spray drying (Huang et al., 2019).

## Conclusions

For high-nickel-type LNCM ternary cathode battery materials, improving energy density, cycle performance, and thermal stability are the focus of future research. The energy band and structure from the material surface and interface need to be analyzed to come up with an improved optimization plan.

High-nickel type NCM ternary cathode materials easily phase change and release oxygen due to the high nickel content. Traditional modification does not essentially solve the structural problems. The core-shell structure promotes the development of high-nickel NCM ternary cathode materials. The high-nickel NCM ternary material with core-shell structure is usually composed of a high-nickel core and a high-manganese shell, which effectively inhibit phase transition and improve cycle performance and thermal stability. The high energy density of the cathode material is ensured. In the core-shell interface of the simple core-shell, the transition metal components cause structural mismatch due to mutations. The final surface of the concentration gradient core-shell still has high Ni content and low Mn content. Under high-strength electrochemical conditions, the structure remains unstable. The full concentration gradient core-shell structure abandons the traditional core-shell boundaries and effectively solves the abovementioned problems. Summarizing the method of synthesizing high-nickel NCM ternary cathode material guides the experiment. In future research, the application of first principles to build a model of synthetic materials for performance calculation can broaden the research ideas and save time and cost.

## Author Contributions

LS and JD wrote the manuscript. ZX, PJ, ZC, and HZ helped to revise the manuscript. All authors contributed to the article and approved the submitted version.

## Conflict of Interest

The authors declare that the research was conducted in the absence of any commercial or financial relationships that could be construed as a potential conflict of interest.
